# Patients’ Perceptions of Experiences of Postoperative Chest Drain Tube Insertion: A Pilot Survey

**DOI:** 10.3390/ijerph20053773

**Published:** 2023-02-21

**Authors:** Agnieszka Kruk, Robert Dziedzic, Sylwia Terech-Skóra, Renata Piotrkowska, Wioletta Mędrzycka-Dąbrowska

**Affiliations:** 1 Department of Surgical Nursing, Medical University of Gdansk, Dębinki 7, 80-211 Gdansk, Poland; 2 Thoracic Surgery Department, Medical University of Gdansk, Smoluchowskiego 17, 80-211 Gdansk, Poland; 3 Department of Anaesthesiology Nursing and Intensive Care, Medical University of Gdansk, Dębinki 7, 80-211 Gdansk, Poland

**Keywords:** drainage, thoracic surgery, patient safety, patient knowledge

## Abstract

Background: Pleural drainage is a routine procedure conducted after thoracotomy and thoracoscopy. It is used to remove air or excess fluid from a pleural cavity and enables proper lung expansion. Essential elements of care provided during hospitalization and treatment include meeting patients’ growing expectations and continually improving quality while optimizing safety. Aim: This study aimed to explore patients’ experiences with pleural drainage after thoracic surgery and their correlation with socio-demographic data. Methods: A pilot survey with an exploratory design was conducted at a large teaching hospital in Poland, in the Department of Thoracic Surgery at the University Clinical Centre in Gdansk. The study involved the analysis of 100 randomly selected subjects with a chest tube drain. A self-designed questionnaire was used to collect social, demographic, and clinical data. Twenty-three questions related to experiences with pleural drainage, ailments, limitations in daily functioning, and security with a chest tube were evaluated using a 5-point Likert scale. Patients completed the questionnaire on the third postoperative day. Results: Individuals fitted with a traditional water-seal drainage system felt safer than those from the digital drainage group (*p* = 0.017). Statistically significant differences were found in the assessment of nursing assistance (*p* = 0.025); the number of satisfied patients was greater in a group of unemployed people. No correlation was found between demographic and social factors and the patients’ sense of security (gender: *p* = 0.348, age: *p* = 0.172, education level: *p* = 0.154, professional activity: *p* = 0.665). Conclusions: Demographic and social characteristics did not significantly affect patients’ sense of safety with chest drainage types. Patients with traditional drainage felt significantly safer than patients with digital drainage. Patient knowledge of pleural drainage management was not satisfactory, with a number of patients indicating a lack of knowledge in this area. This is important information that should be considered when planning measures to improve the quality of care.

## 1. Introduction

Thoracic surgery procedures involve the risk of postoperative complications such as persistent air leaks, subcutaneous emphysema, or accumulation of fluid in the pleural cavity. Pleural drainage is a routine procedure after thoracotomy and thoracoscopy. It is used to remove air or excess fluid from the pleural cavity, enabling proper lung expansion [1,2]. Currently, various drainage systems are used in clinical practice, such as traditional water-seal bottle drainage, Heimlich’s one-way valve, disposable three-chamber drainage, and modern digital drainage [3]. Traditional water-seal bottle drainage has many limitations, as device assessment can be inaccurate and is often subjective. It also requires the patient to adjust their body positioning to properly evacuate fluid and air from the pleural cavity [4].

The first electronic portable drainage system was launched worldwide in 2007. In 2014, an improved system was introduced. This has since been further enhanced with drainage monitoring functions including electronic measurements of possible air and fluid leakage [5]. The operation of this system is similar to that of the water-seal bottle drainage system, but also allows for detailed, round-the-clock monitoring of air leakage parameters, quantification of air leaks, and modifications to the suction power of the drainage [6,7,8]. Patients assessed the digital chest drain positively in terms of mobility and overall comfort. However, there was no clear opinion on the superiority of the digital drainage system over the traditional system in cases of long-term air leakage and patients being discharged with a drainage device implanted [9,10].

Some studies have suggested that large diameter chest tubes (28–32 Fr) may be associated with greater pain. The authors of [11,12] concluded that patients with larger bore tubes experienced moderate to severe pain.

Meeting patients’ growing expectations and continually improving care quality while optimizing safety are all vital elements of care during hospitalization and treatment [13]. Continuous patient education during hospitalization is another of the elements improving the safety of surgical patients [14,15].

The purpose of this exploratory study was to obtain information on patients’ experiences and perceptions of pleural cavity drainage after thoracic surgery, the correlation of these experiences with socio-demographic data collected using a questionnaire, and the application of the results to direct future research in this area.

## 2. Methods

### 2.1. Design

This was a cross-sectional survey study.

### 2.2. Study Procedures

The study was conducted at the Department of Thoracic Surgery at the University Clinical Center in Gdansk in Poland between September 2018 and March 2019. An independent scientific assistant was trained by researchers to conduct the survey using a questionnaire. Patients who still had a pleural drain on the third postoperative day were informed about their voluntary participation in the study. After giving their informed consent, they completed the questionnaire in the room where they were hospitalized. The research assistant was available in the patients’ ward if a question needed clarification. Data concerning the diagnosis and type of operation were cross-checked against the patients’ medical records by the scientific assistant.

### 2.3. Participants

All patients consented to participate in the study. The study group consisted of 100 randomly selected patients. The demographic, social, and clinical features of the respondents are presented in Table 1. The inclusion criteria used were as follows: thoracic surgical treatment, drainage to the pleural cavity, the activity of the patient allowing free movement in the ward, age over 18 years, consent to participate in the study, and a mental condition enabling credible answers. The indication for the procedure was usually a pathologically confirmed non-small cell lung cancer (89%) or suspected malignant disease in the remaining group, such as thymoma or empyema. The predominant procedure used in the study group was lobectomy, performed in 72% of patients. Less commonly used procedures included wedge excision of the tumor at 14%, segmentectomy at 7%, decortication at 5%, and thymectomy at 2%. A 28 Fr or 24 Fr thoracic drain, which was placed during the operation, was used in all patients (Table 2).

### 2.4. Instruments

The research was carried out using a diagnostic survey method and questionnaire. The questionnaire was developed based on our professional experience, clinical observations, and research published in the literature on the subject [1,9,16]. The questionnaire consisted of 2 parts. The first was used to collect demographic and social data (age, gender, level of education, professional activity) and clinical data (date of operation, type of operation, size of a pleural drain, type of a drainage system). The second part consisted of 23 questions, including 17 questions related to experiences with pleural drainage, i.e., ailments and limitations in daily functioning with a chest tube, and 6 questions related to the patient’s sense of security with a chest tube. The questions regarding sense of security with a chest tube are presented in Table 1. The answers were evaluated according to a 5-point Likert scale, where 5 meant “definitely yes” and 1 meant “definitely not”.

### 2.5. Ethical Considerations

The research was approved by the Independent Bioethics Committee for Scientific Research at the Medical University of Gdansk (NKBBN/385/2014).

### 2.6. Statistical Analysis

Qualitative data on the demographic, social, and clinical factors involved were expressed on a nominal or orderly scale. Structure indicators were used for statistical descriptions. The chi-square test of independence and the Mann–Whitney U test were used to verify research questions concerning the relationship between patients’ well-being associated with pleural drainage and demographic, social, and clinical factors. Microsoft Excel (Microsoft, Redmond, WA, USA) and the Statistica 13.1 (Tibco Software, Palo Alto, CA, USA) program were used for statistical analysis. In the calculations obtained, the significance level was assumed to be *p* ≤ 0.05.

## 3. Results

### 3.1. A General Assessment of the Sense of Security of Patients with a Chest Drain

**Figure 1:**
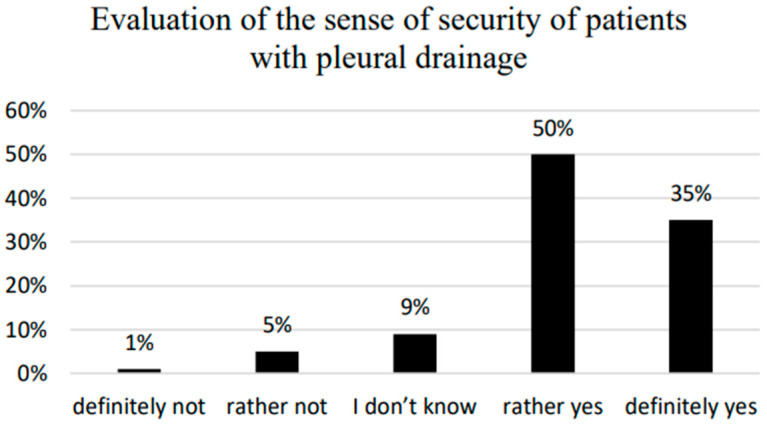
Evaluation of the sense of security of patients with pleural drainage.

In total, 85% of the respondents claimed they felt safe with pleural drainage, whereas about 6% of the respondents stated they did not feel safe (Figure 1). The possible correlation between patients’ overall sense of safety and their demographic and social data was explored (Table 3).

No correlation was found between the patients’ sense of security and their sex, age, level of education, or professional activity. In each group of patients, those who felt safe prevailed.

### 3.2. Clinical Factors and a Sense of Security

The effect of patients’ sense of security on selected clinical aspects and previous experience in this area was examined.

No significant correlation was observed between the patients’ sense of security and the type of procedure (*p* = 0.589) or the size of the drain (*p* = 0.141). Additionally, previous experience with the drainage system did not affect the assessment. Only the type of drainage system significantly affected the sense of security (*p* = 0.017) (Table 4).

**Figure 2:**
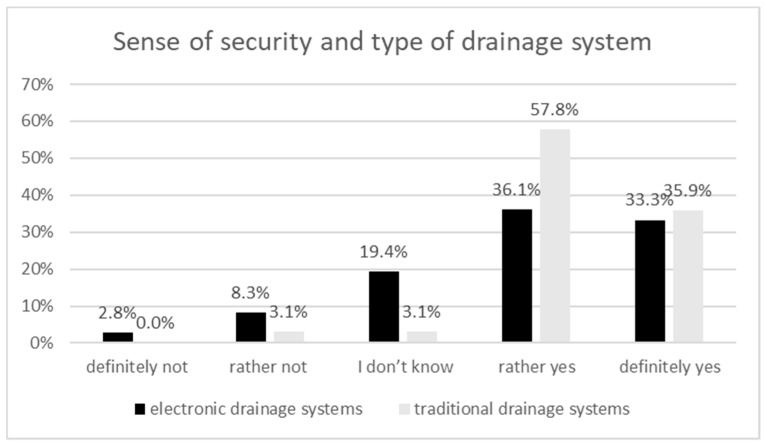
Sense of security and type of drainage system.

Among patients with water-seal drainage systems, the percentage who felt safe was 93.7%, which is 24.3 points more than those with digital drainage systems. About 3% of patients with digital drainage stated, “I definitely do not feel safe”, whereas among patients with bottle-seal drainage, no such response was reported (Figure 2).

**Figure 3:**
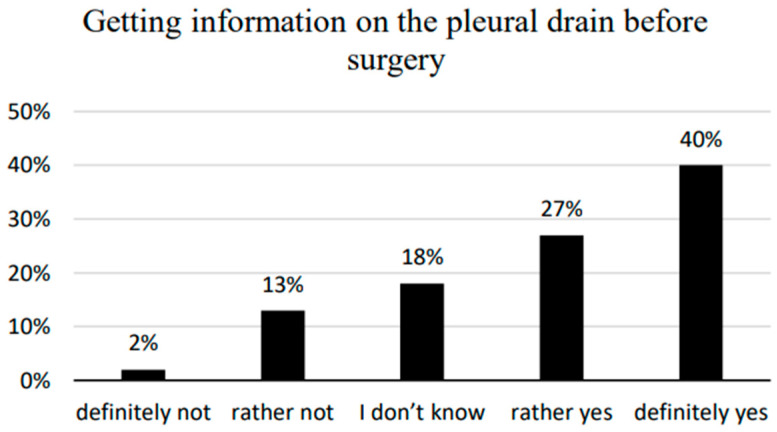
Obtaining information on the pleural drain before surgery.

**Figure 4:**
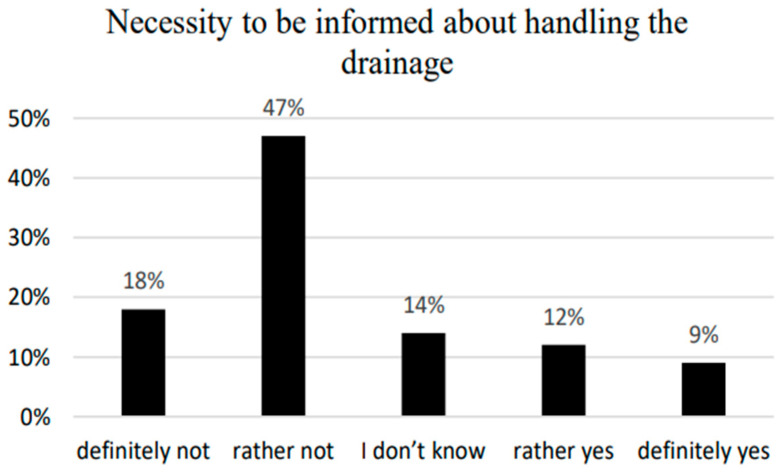
Necessity to be informed about handling the drainage system.

**Figure 5:**
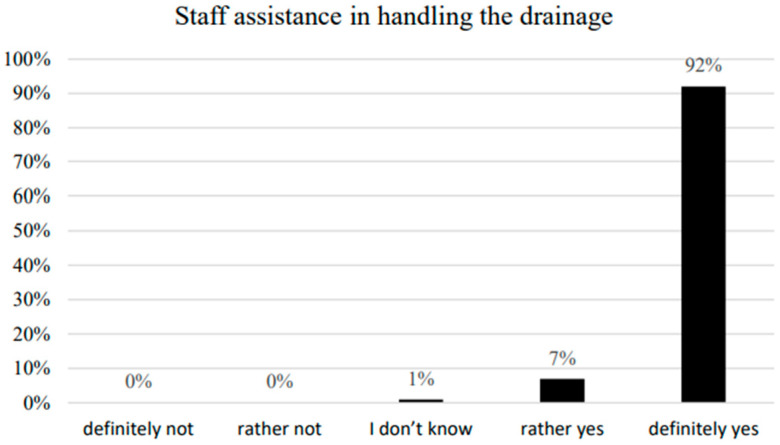
Staff assistance in handling the drainage system.

The respondents were asked if they had received information about the chest drainage system before the procedure, if they felt the need to discuss how pleural drainage is handled, and how they would evaluate nursing assistance in this respect. The majority of the examined patients (67%) stated that they had received information on pleural drainage before the operation. In total, 18% of respondents did not express their opinion on this subject, choosing the answer—”I have no opinion (Figure 3). The majority of patients stated that they did not need further information on handling the drainage system (Figure 4). There was no statistically significant correlation between the need to inform patients about the pleural drain and the demographic and social factors studied. A total of 21% of respondents reported a “Need for information.”; 20.3% of these were patients with traditional drainage systems (13) and 22.2% with electronic drainage systems (8). The differences between the indications were not statistically significant (z = 0.225, *p* = 0.822). A statistically significant correlation was observed only in patients’ assessments of nursing staff assistance and professional activity (*p* = 0.025) (Table 5). Almost all patients (99%) stated that they could definitely depend on the nursing staff to help and none reported not receiving such help (Figure 5).

Among the patients who stated they had received information on the drainage system, 64% were women and 70% were men. A total of 69% were aged 60 or less and 66% were aged 61 or above. A total of 46% had completed primary education and 79% had completed tertiary education, with 75% actively employed and 62% not actively employed.

Regarding the results of the patients who stated they did not need to be better informed about handling the drainage system, 73% were women and 59% were men, with 69% aged 60 or below and 63% aged 61 or above. A total of 54% had completed primary education and 65% had completed higher education with 71% actively employed and 63% who were not actively employed.

Almost all of the women and 88% of the men surveyed said they could definitely count on nursing staff to help them. Among those who answered that they can definitely count on nursing help, 86% were 60 years old or younger, 94% were 61 years old or older, 92% completed primary education, 83% completed higher education, 82% were economically active and 96% were not economically active. Those who were economically active were significantly less satisfied with the nursing staff.

## 4. Discussion

One of the most important human needs is a sense of security, as its absence causes anxiety, fear, and a sense of threat. Maslow’s pyramid of the hierarchy of needs is widely known, and it is not without reason that the need for safety is secondary, just after physiological needs. Therefore, it can be concluded that fulfilling the need for safety is essential for the efficient functioning and development of the whole body [17,18].

Appropriate preparation for surgery is a complex task in which the entire therapeutic team participates. Unfortunately, with the development of medicine and the prevailing trend of shortening a patient’s hospital stay, less and less time is being left for a therapeutic conversation. While preparing for the procedure, medical personnel mainly focus on the technical aspects of treatment and care and pay less attention to emotional support, which has an impact on the patient’s sense of security [19,20].

In recent years, enhanced recovery after surgery (ERAS) protocols have been developed in various surgical fields. One of the pillars of early postoperative care is to reduce stress caused by surgical trauma, thus reducing the number of perioperative complications, accelerating the patient’s return to full activity, shortening the duration of hospitalization, improving the quality of care provided in surgical wards, and reducing healthcare costs [21,22].

This research included a group of patients treated surgically at the Department of Thoracic Surgery, focusing on one characteristic component of postoperative treatment—pleural cavity drainage. Many chest cavity surgery studies have investigated this issue, characterizing the types of drainage systems, their application techniques, and possible complications [2,3,6,7]. Little attention has been paid to the influence of pleural cavity drainage on patients’ functioning and their sense of security in the postoperative period and on the existence of factors which could significantly affect these areas [23].

Currently, education is the key care element enabling success in the treatment, nursing, and rehabilitation of the patient. Acquiring knowledge proves most effective in cases when education begins right after the patient has been admitted to the ward [24,25].

The results of our own research concluded that more than three-fifths of patients examined received crucial pleural cavity drainage information before surgery. More than half of all respondents (65%) stated this knowledge was sufficient, and no additional related information was needed in the postoperative period. Grochans et al. evaluated the preparation of pleural cavity drainage patients for self-care [25]. Their results confirmed that patients with pleural drainage are well-prepared to maintain self-care mainly due to the education provided by nurses. The results reported by Girzelska et al. also show that more than 80% of patients interviewed rated their level of satisfaction with the education provided as either “very high” or “high” [24].

A sense of security is subjective and defined in relation to a given situation. Analysis of the results shows that the majority of patients examined (85%) confirmed feeling safe with pleural cavity drainage during their stay in the ward. The importance of a sense of security, as well as the role of personnel during hospitalization, was studied by Stadnicka et al. [26]. The research conducted in maternity wards determined that for the respondents, the highest value of obstetric care in the perinatal period was from personnel ensuring patients could develop a sense of security. This conclusion was supported by our research, showing that nearly all of the respondents—99%—affirmed being able to receive help from the personnel in the case of problems related to pleural cavity drainage.

When studying factors affecting a sense of security in drainage patients, the clinical aspects were taken into consideration as well: type of surgical access, type of drainage system, and size of the drain. In the present study, the type of drainage system used was shown to be a factor significantly perturbing the sense of security (*p* = 0.017). A study performed by Rathinam et al. on a group of 120 patients and 15 nurses evidenced that digital drainage was well received by said groups, with the technical values of digital equipment receiving the highest ratings [7]. Based on this, one could assume that digital drainage raises patients’ sense of security, although this was not confirmed by our research. This may be due to the fact that patients with a digital drainage system constituted only 36% of the entire focus group. Patients’ opinions require additional research with the aim of understanding factors that reduce the sense of security in a particular group.

The use of digital system drainage is subject to ongoing discussions. Despite the fact that the electronic drainage system is modern, light, has a built-in suction pump, allows for an objective quantified assessment of air leakage or drained fluid volume, and can record volumes over time, the results from the published literature on the routine use of electronic drainage are conflicting. Pompili et al. published their results from a multicenter randomized trial comparing the digital drainage system with the traditional bottle system. They concluded that patients with digital drainage experienced a shorter chest tube placement, a shorter hospital stay, and higher satisfaction scores [27]. On the contrary, another single-center randomized trial published by Gilbert et al. showed no statistically significant impact of type of drainages on hospital stay or duration of drainage [28].

Actions undertaken by healthcare systems to ensure patients’ sense of security during treatment are an essential issue. Systems for ensuring patient security should focus not just on introducing new operation algorithms, but also must strengthen the culture of safety by encouraging communication, trust, and honesty toward patients [6].

## 5. Study Limitations

This study has several limitations. First, a questionnaire of our own design was used to study patients’ experiences, which included a set of questions relating to the specific situations of patients with pleural cavity drainage in place. To the best of our knowledge, no validated measurement tool has yet been developed that addresses the specific problems experienced by patients with pleural cavity drainage. Certainly, based on an interpretive phenomenology, the use of a semi-structured interview would provide an in-depth understanding of the uniqueness of the phenomenon under study. Nevertheless, we included in our study the early postoperative period, in which the patient adapts to functioning with pleural drainage in specific areas, which makes our results important for the nursing team caring for the patient during this period.

Second, the present study is a single-center study conducted at a tertiary hospital in Gdansk, Poland, and the sample size was limited to 100 patients, but as a pilot study it is significant. The decision to use a specific type of drainage at the center where the study was conducted depends on the surgeon and on the patient’s condition. During the study period, digital drainage was used in a small group of patients who were found to have a significantly lower sense of security when using this drainage system. We did not analyze what factors might have influenced this result. In retrospect, we were able to include a question about the use of a modern drainage control system in the questionnaire. Analyzing patients’ perceptions of the electronic drainage system according to age and education level may have influenced the results. Although this is an exploratory study conducted on a limited sample of patients by using a questionnaire of our own design, the results obtained may contribute to obtaining further knowledge in this area.

## 6. Conclusions

Pleural cavity drainage plays an important role in the postoperative period, providing controlled evacuation of fluid and air, and thus ensuring proper lung expansion and proper respiratory physiotherapy. However, there are few studies that address the experience and sense of safety of patients with pleural cavity drainage in the postoperative period.

The questionnaire used may help staff recognize the needs and determine the extent of education needed for patients with pleural cavity drainage to feel safe in the early postoperative period. However, the tool needs further validation and the inclusion of questions about the type of drainage system.

Although most patients received instructions on pleural cavity drainage before surgery and reported no need for additional information after surgery, the results indicate that there is a sizable group of patients with a lack of knowledge in this regard. This is important information that should be taken into account in planning measures to improve the quality of care.

The introduction of electronic drainage systems has significantly improved the safety and quality of nursing staff. It is important that nurses also have knowledge regarding the impact of using modern pleural drainage systems on patients.

## Figures and Tables

**Table 1:** Survey questions and related scores on a 5-point Likert scale used to assess the sense of security in case of patients with a chest tube.

Questions	Multiple Choice Answers and Scores
1. Did you receive information regarding the pleural drainage before the surgery?	1. Definitely yes. 2. Rather yes. 3. I don’t know 4. Rather not 5. Definitely not
2. Do you feel safe with the pleural drainage?	1. Definitely yes. 2. Rather yes. 3. I don’t know 4. Rather not 5. Definitely not
3. Are you afraid of doing particular activities related to the presence of the drainage?	1. Definitely yes. 2. Rather yes. 3. I don’t know 4. Rather not 5. Definitely not
4. What are your biggest concerns related to the presence of the drainage?	1. Accidental disconnection of the drainage set 2. Accidental removal of the drain 3. The presence of substance that is coming out from the drain 4. Moving with drain during activity 5. Damage of the drainage set/breaking the bottle 6. Loosening of the stitches which are supposed to support the drain 7. Other
5. Do you need more information how to handle the drainage?	1. Definitely yes. 2. Rather yes. 3. I don’t know 4. Rather not 5. Definitely not
6. Did you receive nursing staff help each time you had some problems with the drainage?	1. Definitely yes. 2. Rather yes. 3. I don’t know 4. Rather not 5. Definitely not

**Table 2:** Demographic, social, and clinical features of the respondents.

Factor	N	%	Factor	N	%
*Sex*			*Type of surgery*		
Women	44	44%	Thoracotomy	61	61%
Men	56	56%	VATS	39	39%
*Age*			*Type of drainage system*		
18–29	5	5%	Traditional, water-seal bottle	64	64%
30–40	3	3%	Electronic	36	36%
41–50	5	5%	Size of the drain		
51–60	16	16%	24 Fr	5	5%
61 and more	71	71%	28 Fr	95	95%
*Education*			*Experiences with drainage*		
Basic	13	13%	First time	72	72%
Vocational	25	25%	Second or more times	28	28%
Secondary	39	39%			
Higher	23	23%			
*Total*	100	100%	*Employment status*		
			Active professionally	19	19%
			Unemployed	9	9%
			Retired	62	62%
			Pensioner	10	10%
			*Total*	100	100%

Key: VATS—video-assisted thoracoscopic surgery; Fr (the French size)—a drain diameter unit in millimeters multiplied by 3.

**Table 3:** General assessment of the sense of security versus selected demographic and social factors.

Factor	Chi-Square	*p*-Value
Sex	4.453	0.348
Age	6.393	0.172
Level of education	16.81	0.154
Professional activity	9.439	0.665

**Table 4:** Sense of security and selected clinical aspects.

Factor	Chi-Square	*p*-Value
Type of surgery	2.816	0.589
Size of the drain	26.790	0.141
Type of drainage system	12.060	0.017
Previous experience	4.789	0.310

**Table 5:** Receiving information on the drainage system and its handling before surgery, and help from nursing staff in relation to socio-demographic factors.

Factor	Chi-Square	*p*-Value
**Obtaining information on the pleural drain before surgery**		
Sex	0.864	0.930
Age	2.504	0.644
Level of education	12.197	0.355
Professional activity	5.060	0.281
**Need for being better informed about handling the drainage system**		
Sex	7.279	0.122
Age	2.290	0.683
Level of education	10.886	0.539
Professional activity	1.977	0.740
**Assistance from nursing staff**		
Sex	3.574	0.167
Age	3.250	0.197
Level of education	11.966	0.063
Professional activity	7.348	0.025

## Data Availability

Not applicable.
